# Evidence for improved prognosis of colorectal cancer diagnosed following the detection of iron deficiency anaemia

**DOI:** 10.1038/s41598-021-92623-z

**Published:** 2021-06-22

**Authors:** Orouba Almilaji, Sally D. Parry, Sharon Docherty, Jonathon Snook

**Affiliations:** 1 Gastroenterology Unit, University Hospitals Dorset NHS Foundation Trust, Poole, UK; 2 Department of Medical Science and Public Health, Bournemouth University, Bournemouth, UK

**Keywords:** Statistics, Cancer models, Cancer screening, Colorectal cancer

## Abstract

Iron deficiency anaemia (IDA) is common in colorectal cancer (CRC), especially, in right-sided CRC which is known to have an overall worse prognosis. The associations between diagnostic pathway (Bowel Cancer Screening Programme (BCSP), IDA, symptomatic) and tumour side/stage was assessed using logistic regression models in 1138 CRC cases presenting during 2010–2016 at a single secondary-care centre in the UK. In the IDA sub-group, the relationship between CRC stage and the event of having a blood count prior to CRC diagnosis was examined using Bayesian parametric survival model. IDA was found as the only significant predictor of right-sided CRC (OR 10.61, 95% CI 7.02–16.52). Early-stage CRC was associated with both the IDA (OR 1.65, 95% CI 1.18–2.29) and BCSP pathway (OR 2.42, 95% CI 1.75–3.37). At any age, the risk of detecting CRC at late-stage was higher in those without a previous blood count check (hazard ratio 1.53, 95% credibility interval 1.08–2.14). The findings of this retrospective observational study suggest a benefit from diagnosing CRC through the detection of IDA, and warrant further research into the prognosis benefit of systematic approach to blood count monitoring of the at-risk population.

## Introduction

Colorectal cancer (CRC) is the fourth common cancer in the United Kingdom, accounting for 12% of all new cases; and the second common cause of cancer-related death, responsible for about 10% of all cancer deaths in the UK^1,2^. Although the outlook is slowly improving, the 5‐year survival rate for CRC is still relatively poor at 58% because most CRC cases in the UK are diagnosed at late stage^3,4^.

It has been recognized that those with more advanced CRC at diagnosis have a worse prognosis, leading to the development of the TNM staging system for CRC^5^. The association is striking—treated 5-year survival ranges from over 90% for stage I disease down to about 10% for stage IV disease^1,4^. The fact that tumour stage generally increases progressively with time, highlighting the importance of early diagnosis. Unfortunately, CRC may not cause symptoms until the disease is already advanced, and when symptoms do develop there is sometimes reluctance to seek medical advice. The consequence of these delays is that many cases of CRC present at a late stage, with a correspondingly high mortality rate. The focus over recent years has therefore been on early diagnosis by screening of the pre-symptomatic at-risk population^1,6^.

The English Bowel Cancer Screening Programme (BCSP) was developed with the aim of reducing the mortality rate by both earlier detection of CRC and removing polyps which if left untreated might advance to cancer^6^. The BCSP is based on the biennial offer of a faecal occult blood test to all in the population aged 60–74, with a view to colonoscopy if positive.

Bowel cancer screening has been shown to reduce the mortality rate of CRC by about 15% with faecal occult blood testing^7,8^, probably because cases were detected at an earlier stage^1,9^. The proportion of CRCs diagnosed at early stage (I or II) was about 64% for the BCSP in 2017, compared to 47% for GP referrals and 32% for those presenting as emergency admissions^10^. However only about 10% of all CRCs countrywide are detected through the BCSP^11^. The relatively low proportion of screened detected cancers probably relates to a number of factors, including low population uptake (less than 50% in some areas) and limited sensitivity of the initial screening test^9^.

Overall about a third of CRCs occur in the right colon, and these differ in a number of important respects from those found in the left colon^1,12^. Right-sided CRCs tend to present with larger tumours at a more advanced stage, and a correspondingly worse prognosis^12–18^. They are also strongly associated with the finding of iron deficiency anaemia (IDA) at presentation^19–23^, believed to be due to chronic low-grade loss of (iron-rich) blood from the tumour bed, resulting in the slowly progressive depletion of body iron stores. IDA often occurs before any other clinical manifestations of CRC^24^, and as the development of IDA is gradual it may precede the diagnosis of CRC by up to 2 years^25^. This provides a window of opportunity for the detection of CRC earlier in the disease course, particularly for tumours of the right colon, and is the basis of the recommendation for urgent investigation of unexplained IDA in the at-risk population^26,27^.

The study reported here is based on the analysis of a large dataset of patients with CRC presented through different diagnostic pathway at a single centre, and the objectives were twofold. First, to compare the effect of the three major diagnostic routes for CRC—the IDA, BCSP, and symptomatic pathways—on the stage and side of CRC. Second, to explore the scale of the missed opportunity for earlier diagnosis of CRC through the IDA pathway, by assessing the prevalence and results of blood counts prior to CRC diagnosis in the IDA pathway sub-group, and comparing the relationship between prior blood count event and the risk of late-stage disease at diagnosis.

## Methods

This study is a retrospective observational study involved statistical analysis of anonymised secondary clinical data on the Poole Hospital CRC MDT database for the years 2010 to 2016 inclusive. Assuming the smallest effect size (0.1), and significance level = 0.05, the sample size was estimated to be around 967 when power = 80%, and around 1268 when power = 90%. The *Strengthening the Reporting of Observational Studies in Epidemiology* (STROBE) guidelines were used to ensure the reporting of this study. Since this is an observational study, and simply involved the analysis of anonymised secondary clinical data, formal ethical/institutional approvals, consent to participate/publish were not required.

### The association between stage/side and presentation pathway

The first part of this study involved the statistical analysis of 1258 CRC cases. The data was scrutinized in 2018 for the purposes of a service audit and included:

- age at diagnosis;
- sex;
- haemoglobin concentration (Hb) at presentation;
- presentation pathway (IDA, BCSP or symptomatic);
- tumour stage (of the most advanced if synchronous lesions present);
- tumour number, histology and location(s).

Iron deficiency was defined by transferrin saturation < 15% and/or serum ferritin less than the lower laboratory limit of normal at the time of the analysis. The symptomatic group comprised cases with symptoms relating directly to the underlying CRC (other than symptomatic anaemia) that resulted in GP referral or emergency admission to secondary care. Patients with both bowel symptoms and IDA were allocated to a presentation pathway based on which was felt to be the dominant feature—in a few cases this was rather arbitrary, but the allocation was made without knowledge of tumour site or stage.

The diagnosis of CRC was established by standard clinical investigation including colonoscopy, and CT scanning. Tumours were graded according to the simplified TNM staging system^5^ based on the initial radiological appearances, modified in the light of subsequent surgical and pathological findings where available. For the purposes of the analysis, stage I and II CRCs were combined into one category—early stage; whilst stage III and IV CRCs were combined as a second category—late stage. CRCs located at or beyond the splenic flexure were considered left-sided, and those proximal to splenic flexure right-sided. Eight cases had synchronous CRCs, and for the purposes of this study they were considered right-sided if any tumour was proximal to the splenic flexure.

The exclusion criteria were (a) incomplete records (17 cases), (b) second entry due to metachronous CRC (7), (c) other neoplastic diagnoses such as stromal tumours, small bowel carcinoma, neuroendocrine tumours, and anal carcinoma (35), (d) non-incident presentation/diagnosis made at another hospital (27), and (e) diagnosis of CRC on cancer follow-up or as an incidental finding on a scan undertaken for some unrelated reason (34). When no histological confirmation was found, cases were included only if the radiological features were regarded as characteristic of CRC, and they were managed as such clinically.

The effects of age, sex, Hb, and presentation pathway on tumour stage (early/late) or side (left/right) were analysed using simple binary logistic regression models run for each of the predictors separately, with stage or side as the outcome. When any significant association was found (p < 0.05), the predictor was added to a multivariable logistic regression model. Due to correlations with particular presentation pathways (such as in the case of BCSP and age, and IDA and Hb), only simple regression models were built for age and Hb. Statistical methods used to check the validity of the fitted logistic regression models and the goodness of fit are shown in (Table S1, Supplementary Information).

### The association between prior blood test event and stage

The second part of the study involved a detailed assessment of the 171 IDA sub-group from all the 1258 cases dataset. An arbitrary “presentation period” was defined as the 3 months immediately prior to the date of CRC diagnosis. The anonymised data for each subject included whether a blood count had been checked in the 3 years prior to the start of their presentation period, and if so, the date and Hb result for the last blood count in this window. On the basis of published literature regarding temporal changes in blood count prior to the diagnosis of CRC^26^, an arbitrary window of 2 years was taken as the basis of comparison for this study.

A proportional hazards parametric survival model was employed to estimate the effect of previous blood count testing (done/not done) on the onset time of late-stage disease in the IDA sub-group using current status data. Current status data consisted of (a) observation time (CRC diagnosis time) and (b) whether the observation time was smaller or larger than the time to late-stage CRC. Diagnosis time was assumed to be independent of late-stage CRC onset time, and survival time (free of late-stage CRC i.e. diagnosed with early stage CRC) to equal age (in years).

The endpoint of interest was “time to late-stage CRC”. So, if patient *i* was investigated at age Ci and late-stage CRC diagnosed, the time of onset was recorded as the interval [0, Ci]. If early-stage CRC was diagnosed, then the time of late-stage onset was recorded as the interval [Ci, ∞].

The Weibull distribution was specified as the baseline parametric distribution because it allows for constant, increasing, or decreasing hazard rates. To approximate the posterior distribution parameters, four Markov Chain Monte Carlo (MCMC) methods were used (sample size per chain was 1000). As current status data was uninformative, we incorporated prior information into the analysis by extending the parametric model to Bayesian framework. The prior information was based on the following assumptions (a) hazard rates of late-stage disease do not decrease with age and (b) without intervention, all early-stage CRCs would progress to late-stage within 10 years. To incorporate (a) the shape parameter was constrained to be > 1, whilst for (b), we set a maximum possible time equal to the age of the patient plus 10 years instead of the upper end of the interval (∞). Statistical assessments of validity and goodness of fit of the models were based on the method outlined in (Table S1, Figs. S1, S2, Supplementary Information).

The methods used in this study were guided by previous relevant publications^28–32^. R (version 3.6.1) and RStudio (version 1.2.5001) were used to run the statistical analyses and to produce the descriptive statistics, and graphs.

### Ethics declarations

Retrospective analysis of anonymised secondary data, formal research ethics approval was not required.

## Results

### The association between stage/side and presentation pathway

After tidying the database and applying the exclusion criteria, 1138 complete cases were available for detailed analysis. Of these, 90% had histologically confirmed colorectal adenocarcinoma, and most of the remainder had high-grade dysplasia on biopsy, undifferentiated carcinoma, or signet cell carcinoma. As shown in Table 1, almost 70% of cases presented via the symptomatic pathway, with about 15% each through the IDA and BCSP routes. Overall, 45% of cases presented with early-stage disease, and 39% with right-sided tumours.

**Table 1:** Descriptive statistics of the CRC dataset divided by presentation pathway (*IDA* iron deficiency anaemia, *BCSP* Bowel Cancer Screening Programme, and symptomatic group).

		IDA	BCSP	Symptomatic
Number	n (%)	171 (15.0%)	187 (16.4%)	780 (68.6%)
Sex ratio	M/F	1.1	1.5	1.3
Age (years)	Median (Q1–Q3)	78 (71–86)	68 (64–71)	75 (64–83)
Hb at diagnosis (g/l)	Median (Q1–Q3)	89 (80–100)	138 (126–147)	124 (106–140)
Early stage (I + II)	n (%)	89 (52.0%)	115 (61.5%)	310 (39.7%)
Right-sided	n (%)	141 (82.5%)	56 (29.9%)	245 (31.4%)

As anticipated, the BCSP group were more likely to be male, and to be younger. The proportion with right-sided tumours was markedly higher in the IDA group and slightly reduced in the BCSP group.

**Figure 1:**
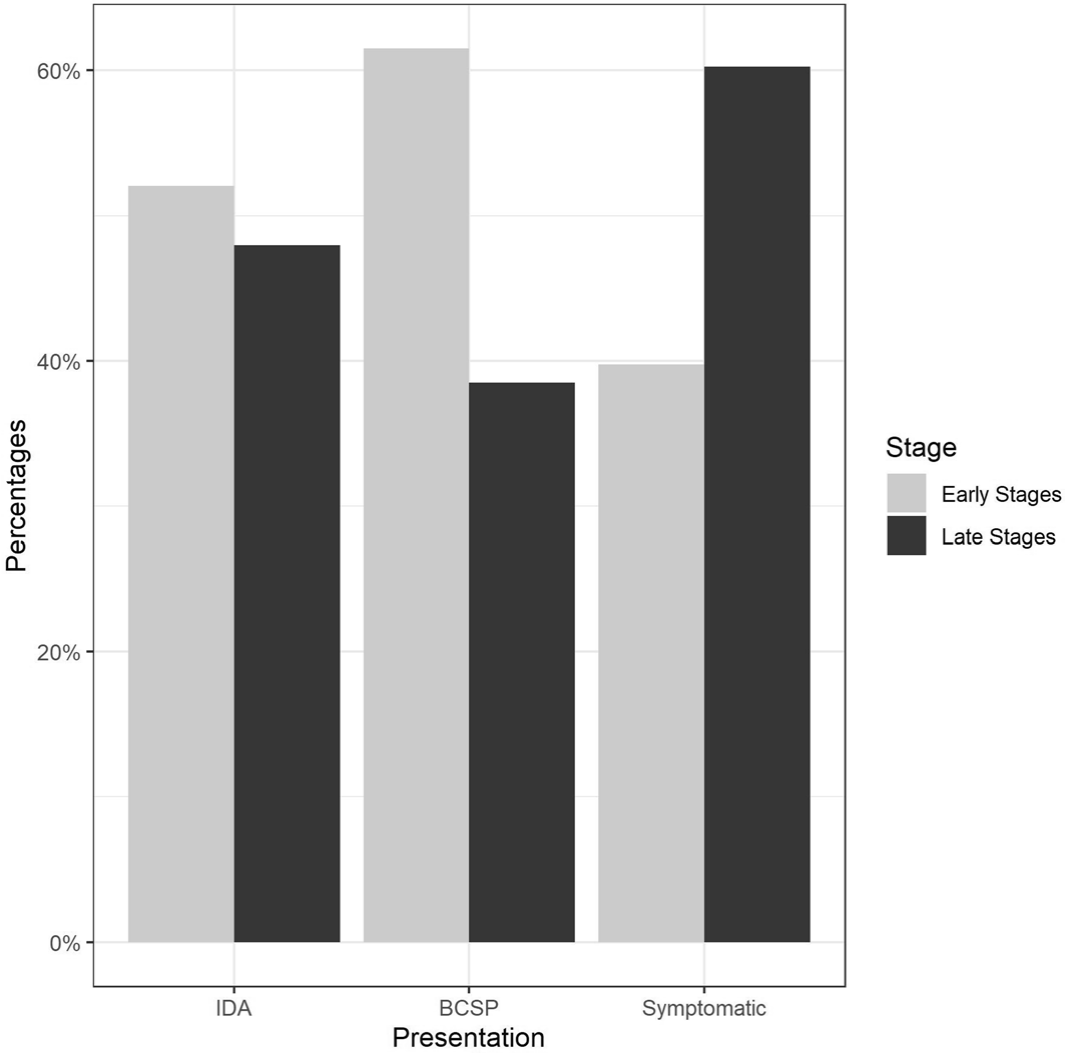
The distribution of tumour stage by presentation pathway.

By crude comparison with the symptomatic group, there was a greater percentage of early-stage CRCs in both of the other groups (Fig. 1).

Four binary logistic regression models were constructed, and their findings are summarised in Table 2.

**Table 2:** Summary of logistic regression analyses showing variables predictive of right-sided CRC (models A–C) and early-stage CRC (model D).

Model	Outcome	Predictor	OR (95% CI)	P value
A	Right-sided CRC	Presentation (IDA)	10.61 (7.02–16.52)	< 0.0001
		Presentation (BCSP)	0.95 (0.67–1.35)	0.78
		Sex (female)	1.94 (1.49–2.53)	< 0.0001
B	Right-sided CRC	Hb (g/l)	0.95 (0.94–0.96)	< 0.0001
C	Right-sided CRC	Age (years)	1.04 (1.03–1.05)	< 0.0001
D	Early-stage CRC	Presentation (IDA)	1.65 (1.18–2.29)	0.003
		Presentation (BCSP)	2.42 (1.75–3.37)	< 0.0001

In *model A*, analysis revealed that sex and presentation pathway were both strongly significant predictors of tumour side. The final multiple binary logistic regression model was therefore constructed according to the formula (left-side CRC as reference category):

The odds of right-sided CRC were about 11 times higher for the IDA pathway than the symptomatic one, whilst the BCSP route was not a significant predictor of right-sided CRC. CRCs were 94% more likely to be right-sided in females compared to males.

In *model B*, Hb was found to be a very significant negative predictor of right-sided CRC—for each unit (g/l) decrease in Hb, there was about a 5% increase in the odds of right-sided CRC.

*Model C* showed that age is also a very significant positive predictor of right-sided CRC—for each rising year of age, the odds of right-sided CRC increased by about 4%.

In *model D*, statistical analysis showed that only presentation pathway was a significant predictor of early-stage CRC. The association between tumour side and stage is not statistically significant (p = 0.07). The final binary logistic regression model was therefore constructed according to the formula (late stage as reference category):

The findings indicate that IDA was a significant positive predictor of early stage CRC. Results also show CRCs presenting through the IDA and BCSP routes are 65% and 142% respectively more likely to be diagnosed at early stage, as compared to the symptomatic pathway. Statistical assessment of validity and goodness of fit of the logistic regression models was satisfactory (based on the criteria outlined in Table S1—Supplementary Information).

### The association between prior blood test event and stage

**Figure 2:**
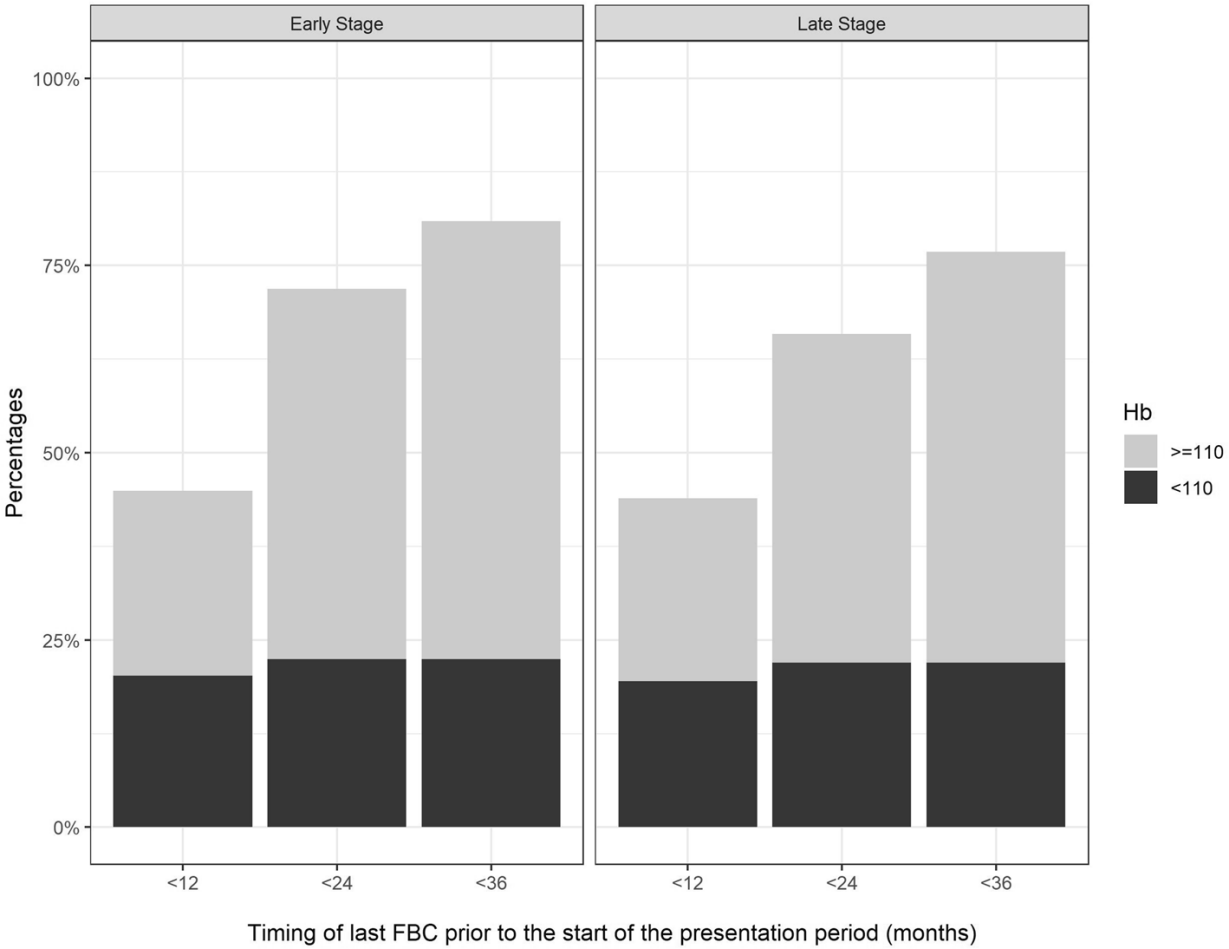
The cumulative percentage prevalence of blood count checks in the 12, 24 and 36 months prior to the diagnosis of CRC in the IDA group, sub-divided according to the tumour stage (early/late) and Hb result (g/l).

Figure 2 shows the cumulative percentage prevalence of blood counts over the 3 years prior to the presentation period for CRC, for the 171 cases presenting via the IDA pathway.

In the 2 years prior to diagnosis of CRC, 31% of did not have a record of any blood count, and a further 22% had an abnormally low blood count (Hb < 110 g/l) which did not result in immediate referral. Most of these abnormal results were recorded in the 12 months prior to the presentation period.

Descriptive statistics for the IDA group broken down by the result of the last blood count in the 2 years prior to the presentation window of CRC are shown in Table 3. There were trends towards those with ‘blood test not done’ being younger, more likely to have right-sided CRC, and less likely to have early-stage disease.

**Table 3:** Descriptive statistics for the IDA group (n = 171) by outcome of the last blood count in the 2 years prior to presentation with CRC.

		Hb > 110 g/l	Hb < 110 g/l	Not done
Number	n (%)	80 (47%)	38 (22%)	53 (31%)
Sex ratio	M/F	1.5	0.7	1.1
Age (years)	Median (Q1–Q3)	78 (75–85)	83 (77–88)	73 (66–83)
Hb at diagnosis (g/l)	Median (Q1–Q3)	94 (85–102)	84 (71–92)	87 (74–99)
Early stage	n (%)	44 (55.0%)	20 (52.6%)	25 (47.2%)
Right-sided	n (%)	65 (81.2%)	30 (78.9%)	46 (86.8%)

**Figure 3:**
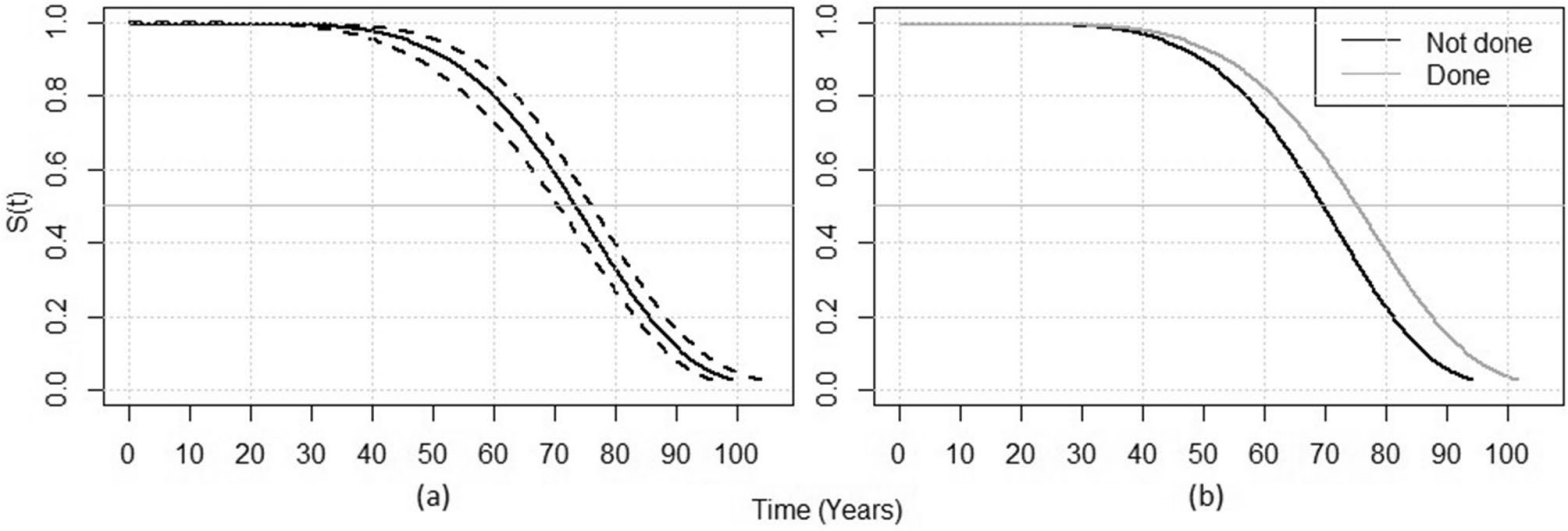
Posterior survival distributions for the IDA group, showing (**a**) the baseline survival probability at any given time S(t), with dashed lines representing the credibility interval, and (**b**) the survival probabilities for sub-groups categorised by whether a blood count was done in the two years prior to the presentation window.

Bayesian Weibull regression showed that the posterior baseline survival distribution of IDA patients with early-stage CRC at diagnosis (i.e. not having reached late-stage disease) decreased with increasing age. This survival figure fell from 80% at age 60 to about 35% at age 80 (Fig. 3a); a. Analysis also revealed that having a prior blood test (regardless of result) was significantly related to time to late-stage disease (mean (sd): 0.66 (0.18), 95% credibility interval 0.46–0.93). Looked at the other way, the hazard ratio for detecting CRC at late-stage was 53% higher in those without a previous blood count (1.53, 95% credibility interval 1.08–2.14).

The posterior median onset time of late-stage CRC in those with a blood count in the preceding 2 years was 75 years (95% credibility interval 72–78). This was 5 years later than the median onset time of 70 years of age (95% credibility interval 65–74) for those without a blood count. This implies that at a given age, the probability that CRC is detected at an early-stage is higher in those with a previous blood count (Fig. 3b).

## Discussion

Our results demonstrate that during the 7-year study period, just over 30% of CRCs were diagnosed via either the IDA or BCSP pathway, with similar numbers in each. Comparison with national data reveals a similar proportion of early-stage disease diagnosed through the symptomatic pathway, at around 40%^10^. The figure for the BCSP pathway is slightly lower than the national figure (62% v 64%), but this may reflect the higher proportion of right-sided cancers detected (30% v 23%)^9,10^.

We have confirmed previous observations that IDA is strongly associated with right-sided CRC, but the striking finding from our study is that diagnosis through a contemporary IDA pathway has the potential to downstage the disease, as previously demonstrated for the BCSP pathway^9^. This is in contrast to reports in the literature suggesting that IDA is a marker of poor prognosis in CRC^22,23,33,34^. The reasons why our findings differ from those of some historical studies may include issues of confounding and diagnostic delay. Firstly, the risk of confounding arises from the strong association between IDA and right-sided CRC—a pattern of disease which is associated with later diagnosis at a more advanced stage, and a correspondingly poorer prognosis^12–18^. Secondly, the diagnosis of CRC through the IDA pathway has been beset by delays resulting in late diagnosis of CRC and so a poor prognosis^35–39^.

In years past major delays at three points in the pathway of CRC diagnosis through the detection of IDA were all too common, and the cumulative effect of these delays may have been a major contributor to the historical association of IDA with poor prognosis in CRC^35–39^. The first is confirmation of IDA on a blood test—a particular issue as even severe anaemia may not cause appreciable symptoms. The second is lack of awareness of the significance of IDA as a marker of underlying malignancy, and therefore of the importance of swift referral for investigation. The third is the time between referral and an adequate diagnostic examination of the (right) colon. Survival in anaemic CRC appears to be inversely related to this last delay^39^.

Various developments over recent years have had a major bearing on these delays. Firstly, routine blood count checks in the at-risk population have become much more frequent and widespread—the rate of blood count testing increased progressively in the UK between 2000 and 2015, from approximately 160 to 430 per 1000 population per annum^40^.

Secondly, much has been done to accelerate the referral and comprehensive investigation of patients found to have IDA, particularly those at risk of CRC. This includes education in primary care, national guidelines encouraging fast-track referral^26,27^, and the development of dedicated IDA triage services in secondary care—such as the IDA Clinic at Poole^41,42^, which was incidentally operational throughout the years of this study. Finally, gastroenterology speciality groups have introduced quality initiatives to improve the diagnostic yield of investigation, particularly in the right colon^43^.

The strengths of this study are the novelty of examining the association between the event of having prior blood count check and the CRC stage in IDA patients, and inclusion of a BCSP CRC group as a positive control. Limitations include the uncertain applicability of a single centre experience to other populations, and being a retrospective analysis, our inability to control the size of the study subgroups or to incorporate other variables that could impact the prognosis in CRC. In fact, the major potential constraint of the study was the use of stage/side as the only markers of prognosis in CRC. We feel however that this methodology is justified because the link between stage/side and prognosis is so strong^1,4^, and this view is supported by the results for the BCSP group, which fit well with the established improvement in prognosis with this programme^7–9,44^. Nonetheless, further studies are clearly warranted to corroborate the findings.

With correction for confounding and a reduction in diagnostic delays, our results suggest that CRC in the right colon may be detected at earlier stage with a correspondingly better prognosis. This observation strengthens the case for the inclusion of monitoring for IDA in the repertoire of screening approaches for the early diagnosis of CRC. Currently however there is no systematic process for routinely checking blood counts in the at-risk population, despite the universal presence of the necessary laboratory infra-structure.

Bearing in mind that the development of IDA is a gradual process prior to the diagnosis of CRC, our results suggest that there may be scope for further improvement in how we screen for bowel cancer. Of those diagnosed with CRC via the IDA pathway, some 31% had not had a blood count in the 2 years prior to diagnosis, whilst a further 22% had a low blood count—that in retrospect may perhaps have been indicative of undiagnosed CRC. Our results also suggest that the median onset age of late-stage CRC in those with a previous blood count is about 5 years older than in those without, so that for a given age, the proportion of CRCs detected at early stage is higher. This is an interesting observation for which there are various possible explanations, but we feel that confounding is perhaps the most likely—individuals who avoid medical care are inherently less likely to have a blood test, and also less likely to present early with their undiagnosed CRC.

Nevertheless, we feel that a strong case can be made for formally recommending a blood count test on perhaps an annual basis in the at-risk population—with follow-up iron studies for those with detected anaemia. Blood count checks have an advantage over the current CRC screening modalities of stool testing or sigmoidoscopy in being more acceptable to many people. However, the recommendation would be to introduce blood count checks as a screening test complementary to the current BCSP, not an alternative. The logic to this is that IDA screening would be expected to predominantly detect right-sided CRC, whilst the current BCSP predominantly targets left-sided CRC, with the suspicion that it may be less effective at picking up right-sided lesions^9,44^.

In conclusion, our findings suggest prognostic benefit from diagnosing CRC through the detection of IDA, and that IDA screening is currently sub-optimal. These observations strengthen the case for a systematic approach to blood count monitoring of the at-risk population.

## Supplementary Information

Supplementary Information.

## Data Availability

Data and code available on reasonable request.

## Abbreviations

CRC: Colorectal cancer
IDA: Iron deficiency anaemia
Hb: Blood haemoglobin concentration
GP: General Practitioner
BCSP: Bowel Cancer Screening Programme
OR: Odds ratio
CI: Confidence interval
