# Supplementary material for: Evidence for improved prognosis of colorectal cancer diagnosed following the detection of iron deficiency anaemia
Source: Sci Rep. 2021 Jun 22;11:13055. doi: 10.1038/s41598-021-92623-z (PMC8219720; doi:10.1038/s41598-021-92623-z)

**Evidence for improved prognosis of colorectal cancer diagnosed following the detection of iron deficiency anaemia**

Orouba Almilaji ^1 2^, Sally D Parry ^1^, Sharon Docherty ^2^, Jonathon Snook ^1^

^1^ Gastroenterology Unit, University Hospitals Dorset NHS Foundation Trust, Poole, UK

^2^ Department of Medical Science and Public Health, Bournemouth University, Bournemouth, UK

**Correspondence:** Orouba Almilaji

Department of Medical Science and Public Health

Bournemouth University

Bournemouth, UK

E-mail: [oalmilaji@bournemouth.ac.uk](mailto:oalmilaji@bournemouth.ac.uk)

**Supplementary Information**

**Table 1** Statistical assessment methods employed

| **Analysis** | **Methods** |
| --- | --- |
| The association between stage/ side and presentation pathway | Smoothed scatter plot, Cook’s distance and standardised residual errors, variance inflation factor, Akaike information criterion, analysis of variance, χ2 test and pseudo R2 were used to check the validity of the fitted logistic regression models and the goodness of fit. |
| The association between prior blood test event and stage | To inspect the Bayesian Weibull model fit, we examined the baseline distribution by plotting it against the semi-parametric estimate to see whether there were systematic deviations for the chosen parametric distribution from the semi-parametric (Fig. 1). Trace plots were used to confirm that the model converged to the target distribution (Fig. 2), and that the sampled values for each parameter in the chain were overlapping with values close to 1. |

**Fig. 1** Demonstration of the baseline distribution against the non-parametric estimate and other parametric families


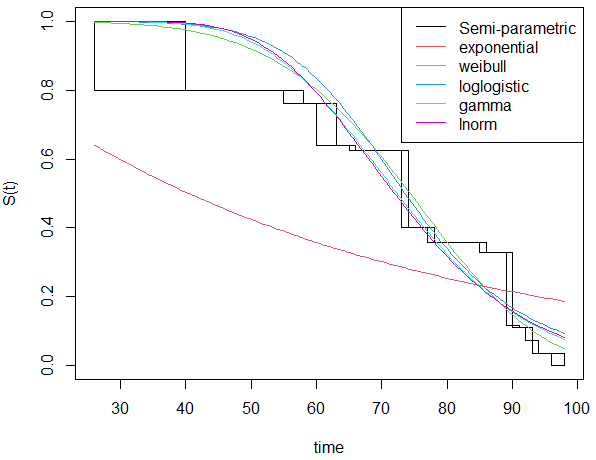


**Fig. 2** Demonstration of the trace plots and marginal density estimates of the posterior samples


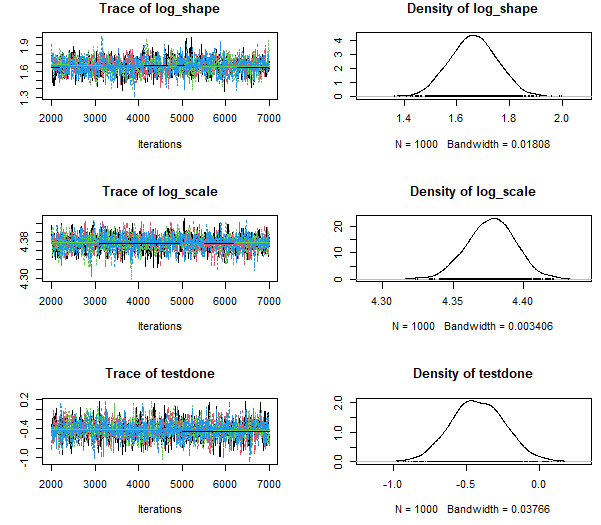

Supplement: Supplementary file 1 — Supplementary Information. [file 41598_2021_92623_MOESM1_ESM.docx]
